# Scent of a break-up: phylogeography and reproductive trait divergences in the red-tailed bumblebee (*Bombus lapidarius*)

**DOI:** 10.1186/1471-2148-13-263

**Published:** 2013-12-02

**Authors:** Thomas Lecocq, Simon Dellicour, Denis Michez, Patrick Lhomme, Maryse Vanderplanck, Irena Valterová, Jean-Yves Rasplus, Pierre Rasmont

**Affiliations:** 1 Laboratoire de Zoologie (Research Institute of Biosciences), University of Mons, Place du Parc 20, B-7000 Mons, Belgium; 2 Evolutionary Biology and Ecology, Université Libre de Bruxelles, Avenue FD Roosevelt 50, B-1050 Brussels, Belgium; 3 Institute of Organic Chemistry and Biochemistry, Academy of Sciences of the Czech Republic, Flamingovo nam 2, CZ-166 10 Prague, Czech Republic; 4 Institut national de la recherche agronomique, UMR 1062 Centre de Biologie pour la Gestion des Populations, CS 30 016, F-34988 Montferrier/Lez cedex, France

**Keywords:** Phylogeography, Reproductive traits, Genetic differentiation, Bumblebees

## Abstract

**Background:**

The Pleistocene climatic oscillations are considered as a major driving force of intraspecific divergence and speciation. During Ice Ages, populations isolated in allopatric glacial refugia can experience differentiation in reproductive traits through divergence in selection regimes. This phenomenon may lead to reproductive isolation and dramatically accentuates the consequences of the climatic oscillations on species. Alternatively, when reproductive isolation is incomplete and populations are expanding again, further mating between the formerly isolated populations can result in the formation of a hybrid zone, genetic introgression or reinforcement speciation through reproductive trait displacements. Therefore changes in reproductive traits driven by population movements during climatic oscillations can act as an important force in promoting pre-zygotic isolation. Notwithstanding, divergence of reproductive traits has not been approached in the context of climatic oscillations. Here we investigate the impact of population movements driven by climatic oscillations on a reproductive trait of a bumblebee species (*Bombus lapidarius*). We characterise the pattern of variation and differentiation across the species distribution (i) with five genes (nuclear and mitochondrial), and (ii) in the chemical composition of male marking secretions (MMS), a key trait for mate attraction in bumblebees.

**Results:**

Our results provide evidence that populations have experienced a genetic allopatric differentiation, in at least three main refugia (the Balkans, Centre-Eastern Europe, and Southern Italy) during Quaternary glaciations. The comparative chemical analyses show that populations from the Southern Italian refugium have experienced MMS differentiation and an incipient speciation process from another refugium. The meeting of Southern Italian populations with other populations as a result of range expansion at a secondary contact zone seems to have led to a reinforcement process on local MMS patterns.

**Conclusions:**

This study suggests that population movement during Quaternary climatic oscillations can lead to divergence in reproductive traits by allopatric differentiation during Ice Ages and by reinforcement during post-glacial recolonization.

## Background

Historic events such as climatic and topographic changes have triggered current patterns of biodiversity [1,2]. The Pleistocene glaciations, in particular, are considered as a major driving force of intraspecific divergence, diversification and speciation (e.g. [3-5]). The Pleistocene epoch experienced important climatic oscillations [6] that have deeply modified the geographic range of temperate species within the Palaearctic region (e.g. [7]). During the Ice Ages, changes in species distribution occured as the ice sheets advanced to reach climatically more favourable regions, called refugia [8,9]. At the end of each Ice Age, some populations expanded from these refugia to recolonize previously glaciated areas [5,10].

Geographic isolation between conspecific populations (e.g. between refugia) is recognised to play an important role in allopatric speciation (e.g. [11,12]). When gene flow has been limited, or absent, among populations, different or identical alleles can become fixed depending upon the forces of natural selection and/or genetic drift [13,14]. Thus genetic divergence occurs over a long period, imparting reproductive isolation and possibly leading to speciation [11]. When reproductive isolation is incomplete and formerly isolated lineages meet as a result of range expansion, further mating between the populations produces hybrids, resulting in the formation of a hybrid zone (e.g. [15,16]), genetic introgression (e.g. [17]) or reinforcement speciation through pre-zygotic isolation mechanisms (e.g. [18-20]).

Sexual selection is well recognized for its important role in promoting speciation through its variation among allopatric populations [20-23], either through the local features of a mating signal [24], or different signals in different environments that confer local adaptations [20,25]. Divergence in mating preferences that parallel divergence in mating signals give rise to discrimination against foreign mates, and can ultimately result in reproductive isolation and speciation ([24-27] but see also [28]). This phenomenon may dramatically increase the consequences of climatic oscillations on species. Notwithstanding, the divergence in reproductive traits has not been approached in the context of climatic oscillations and migration of populations. Previous studies have mainly focused on the consequences of climatic oscillations on genetic traits (e.g. [10,15,16]) while the consequences on reproductive traits and comprehensive integrative analyses of reproductive traits, geographical variation, and genetic differentiation are lacking.

**Figure 1:**
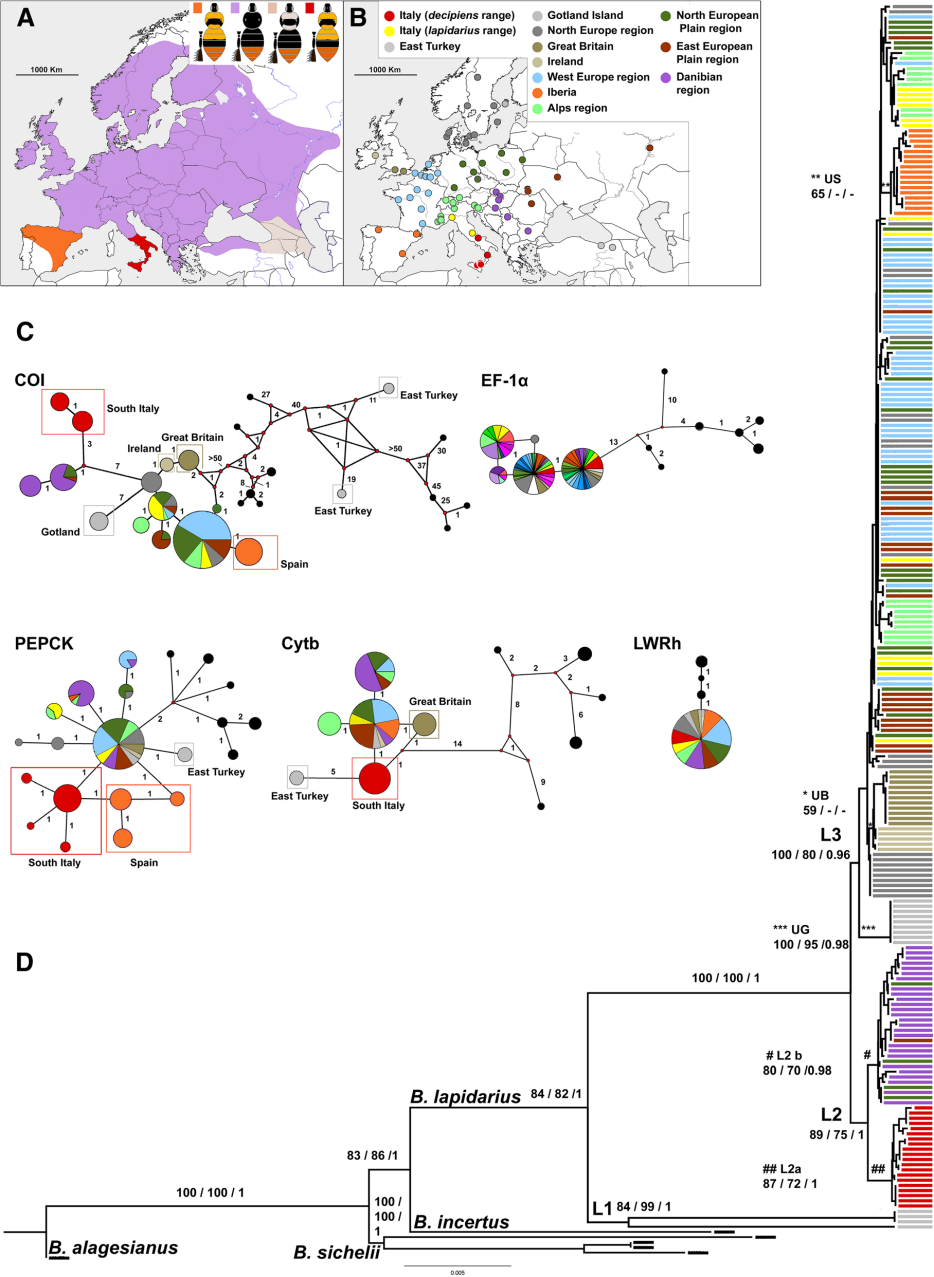
**Distribution and phylogenetic relationship of *B. lapidarius*. A**: Colour pattern distribution, all geographic zones are the *B. lapidarius* geographic area; **B**: Map of sampling, colour of points refers to geographic zones. **C**: Median-joining network of haplotypes based on *COI*, *Cytb*, EF1α, *PEPCK* and *LWRh*. Circle sizes are proportional to frequencies of haplotypes. Colours of haplotypes refer to geographic area (Figure 1B). Red points on lines, undetected/extinct intermediate haplotype states. Numbers on lines represent the number of mutation(s) between two close haplotypes. Black circles are outgroups. **D**: Neighbour-joining tree based the combined molecular data matrix (*COI*, *Cytb*, EF1α, *PEPCK* and *LWRh*), value above branches are Neighbour-joining bootstrap values (only values > 50 are shown)/ maximum likelihood bootstrap values (only values > 70 are shown)/Bayesian posterior probabilities (only values > 0.95 are shown). Colour labels of branches refer to geographic area (Figure 1B).

Here we investigated the phylogeography, population structure, and geographical variation of a reproductive trait (male marking secretion, MMS) in the red-tailed bumblebee, *Bombus lapidarius.* Bumblebees are an excellent system for exploring historical biogeographic patterns of Holarctic groups and the evolution of the courtship signal (e.g. [23,29]). These bees live in the coldest areas inhabited by insects and have been able to recolonize areas depopulated by Ice Ages in the last three million years [29]. Their early historical biogeography (from Eocene-Oligocene to Miocene) has already been explored [29] while their recent history (from Pliocene to Holocene) has received comparatively far less attention (e.g. [3]). *B. lapidarius* is a widespread temperate West-Palaearctic bumblebee that includes several subspecies which ex-hibit different colour patterns (Figure 1A, [30]): (i) *B. lapidarius lapidarius* in the European plains, Balkans and West Anatolia, (ii) *B. lapidarius decipiens* in the Iberian and South Italian peninsulas, (iii) *B. lapidarius caucasicus* and *B. lapidarius eriophorus* in N.E. Anatolia and the Caucasus, and (iv) *B. lapidarius atlanticus* in the Moroccan Atlas.

The courtship signal of bumblebee males includes both behavioural and chemical features (see [31] for a review). Here, we focus on the most commonly studied trait, the male marking secretions (MMS). Most bumblebee males patrol along paths where they scent-mark objects with their MMS that attract conspecific virgin females [32]. The MMS consist of a complex mixture (mainly aliphatic compounds) with several major components and with intraspecific variation (e.g. [33,34]). MMS are produced *de novo* by cephalic labial glands [35] from saturated fatty-acids by the action of species-specific esterases, desaturases and reductases in cephalic labial glands [35,36].

In this study, we used a phylogeographic approach based on five genes (nuclear and mitochondrial) along with comparative chemical analyses of MMS to determine the consequences of historic events on the chemical composition of male courtship signals. Our purposes were (i) to infer the phylogeographic pattern of this widespread European pollinator, (ii) to detect a putative divergence of MMS across its distribution range, and (iii) to compare the phylogeographic pattern and the reproductive trait variation pattern.

## Results

### Genetic divergence and phylogeography of the red-tailed bumblebee

#### *Patterns of sequence variation*

**Figure 2:**
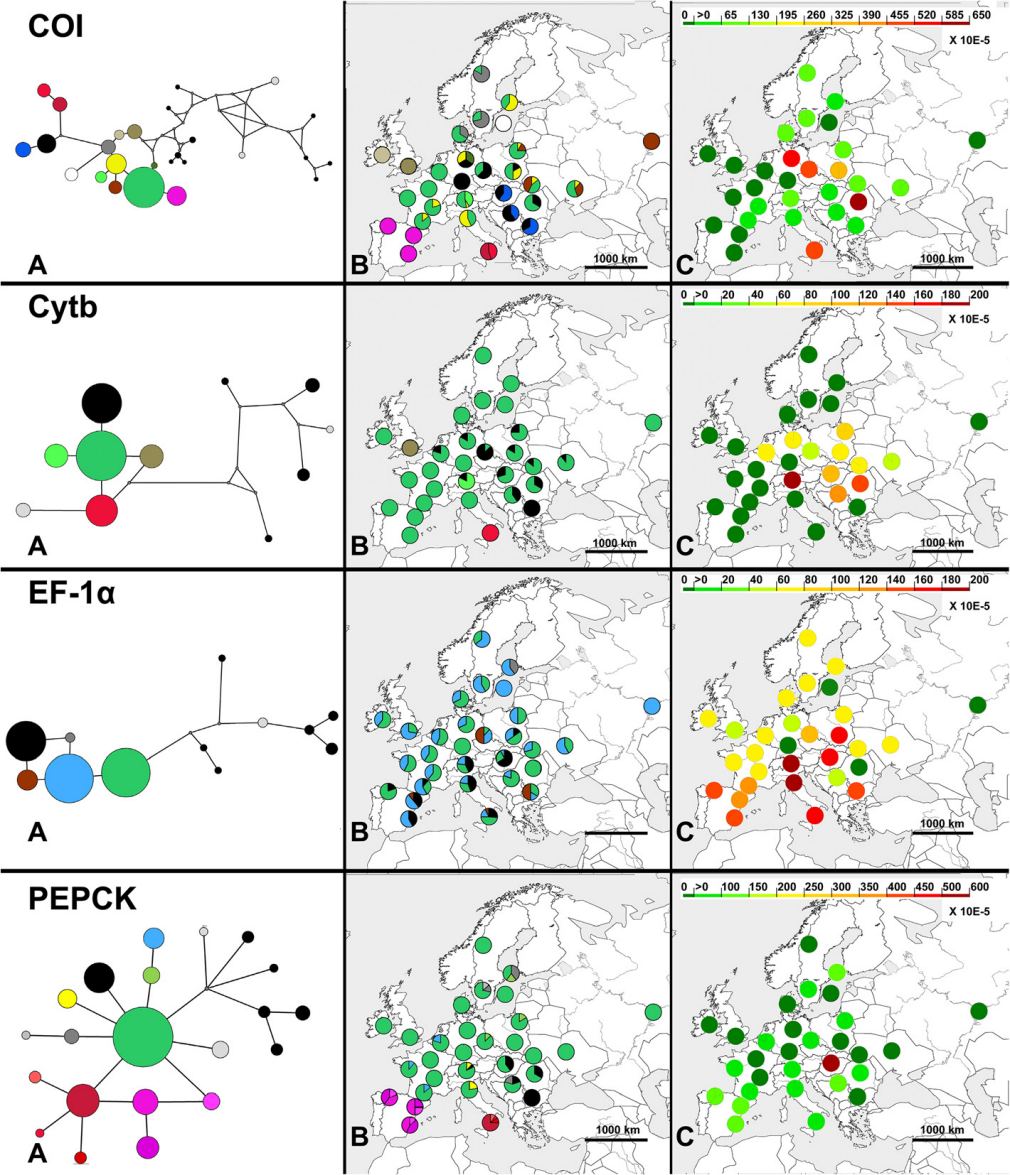
**Geographic distribution of genetic diversities in *B. lapidarius*.** In the left column median-joining network of haplotypes based on *COI*, *Cytb*, EF1α, and *PEPCK* are coloured by haplotypes. In the centre column maps of haplotype frequency for each geographic groups based on *COI*, *Cytb*, EF1α, and *PEPCK* have the colours of pie charts as for haplotype colour (left column). In the right column maps of nucleotide diversity for each geographic group based on *COI*, *Cytb*, EF1α, and *PEPCK*, Colours of pie charts refer to coloured intensity scale.

The final molecular dataset spanned 3933 aligned nucleotides: 1056 bp from *COI* (184 parsimony informative sites [PIS]), 465 bp from *Cytb* (37 PIS), 791 bp from *EF-1α* F2 copy containing a ~200 bp intron (9 PIS), 711 bp from *LWRh* (2 PIS), and 910 bp from *PEPCK* (11 PIS). Alignments did not require additional indels. *LWRh* was monomorphic across all *B. lapidarius* populations while other loci were polymorphic (Figure 1C). Therefore, we excluded the *LWRh* from the following results. In total, 16 unique haplotypes were observed for *COI*, 7 for *Cytb*, 6 for *EF-1α*, and 16 for *PEPCK* among 244 European and East Turkish individuals (Figure 1C). For geographic groups, haplotype diversity ranged from 0 to 0.8 and nucleotide diversity ranged from 0 to 0.006 (Additional file 1, Figure 2). Our results displayed endemic haplotypes or haplogroups from (Figures 1C, 2): (i) Gotland Island, Great Britain, Ireland, Southern Italy, East Turkey and Spain for *COI*; (ii) Great Britain, East Turkey, and Southern Italy for *Cytb*; (iii) Southern Italy, East Turkey, and Spain for *PEPCK*.

#### *Phylogeny and haplotype relationships*

All phylogenetic analyses and MJM networks performed on the same genetic dataset led to quite similar topologies (Figure 1C, see also supplementary trees [TreeBASE: ID S14299]). Phylogenetic analyses based on *EF-1α* did not show genetic divergence between geographic groups and failed to support strongly the relationship with outgroups. In the same way, despite the distinctive haplotypes of some geographic groups, phylogeny based on *PEPCK* failed to resolve relationships between haplogroups while trees based on *COI* and *Cytb* resolved the relationship between haplogroups. Phylogenetic analyses based on nuclear genes (*EF-1α* + *PEPCK*) failed to resolve the relationship between genetic groups while analyses based on mitochondrial genes (*COI* + *Cytb*) and all genes combined (*COI* dominated the phylogenetic signal) resolved the relationship between genetic groups. Phylogeny based on all genes combined showed a large-scale structuring in main groups that have a predominantly non-overlapping geographic distribution (Figure 1D). *B. lapidarius* is split into three major lineages: an East Turkey lineage (L1), a Southern Italy and SE European lineage (L2), and a large group including all other European populations (L3) (Figure 1D). The lineage L2 included two sub-lineages: a Southern Italian lineage (L2a) and a SE European lineage (L2b). Inside L3, three distinct geographic groups were distinguished: a Gotland group (UG), a British Isles group (UB), and a Spanish group (US) but they were poorly supported. However there is evidence for divergence between islands, peninsulas and other populations and for maintenance of distinct genetic structure over time. Due to the large genetic divergence of the East Turkey lineage (L1), its very restricted geographic distribution, and the lack of MMS data, we chose to exclude these samples from our analyses of population structure.

#### *Divergence times*

Based on the *COI* data, the estimated divergence between East Turkish (L1) and European populations (L2 + L3) was within a range of 0.49 - 9.56 mya, with a mean of 2.81 mya and median of 1.32 mya (ESS: 222.99) while estimated divergence between European haplogroups (L2 versus L3) was within a range of 0.03-0.96 mya, with a mean of 0.28 mya and median of 0.13 mya (ESS: 231.21). Correspondingly, average simple pairwise divergence (i) between L1 and L2 + L3 was 9.68% or ~ 4.9 mya based on the 2% divergence per million years clock commonly applied to insect mitochondrial DNA [37], and (ii) between L2 and L3 was 1.01% or ~ 0.51 mya.

#### *Population structure*

Geographic structuring among *COI*, *Cytb* and *PEPCK* haplotypes within European populations was significantly supported by AMOVA results (global Φ_ST_ statistic = 0.87 (*COI*), 0.79 (*Cytb*), 0.61 (*PEPCK*), p-value < 0.01). These results suggested a potentially low level of gene flow between several adjacent populations. *EF-1α* and *LWRh* datasets were discarded from SAMOVA due to their poor genetic structuring (global Φ_ST_ statistic = 0.35, p-value < 0.01 for EF-1 α and p-value > 0.01 for *LWRh*). In SAMOVA, the Φ_CT_ value increased asymptotically with increasing number of groups, levelling out at: seven groups for *COI* (Φ_CT_ = 0.87, Φ_ST_ = 0.93, Φ_SC_ = 0.60) with local Φ_CT_ maximum at five groups (Φ_CT_ = 0.85, Φ_ST_ = 0.94, Φ_SC_ = 0.57) and three groups (Φ_CT_ = 0.81, Φ_ST_ = 0.94, Φ_SC_ = 0.66), five groups for *Cytb* (Φ_CT_ = 0.89, Φ_ST_ = 0.90, Φ_SC_ = 0.06), nine groups for *PEPCK* (Φ_CT_ = 0.82, Φ_ST_ = 0.80, Φ_SC_ = -0.09) but above five groups (Φ_CT_ = 0.78, Φ_ST_ = 0.81, Φ_SC_ = 0.13), additional groups represented single sampling sites making little biological sense. All SAMOVA clustering (based on *COI*, *Cytb* and *PEPCK*) split the Southern Italian populations and most of the SE European populations from other populations while only *PEPCK* clustering split Spanish populations from other populations (Additional file 2). Clustering based on mitochondrial genes split some insular populations (Additional file 2). These SAMOVA results were quite congruent to geographic groups observed in MJN and phylogenetic results.

#### *Inter-individual genetic distance and genetic variability*

**Figure 3:**
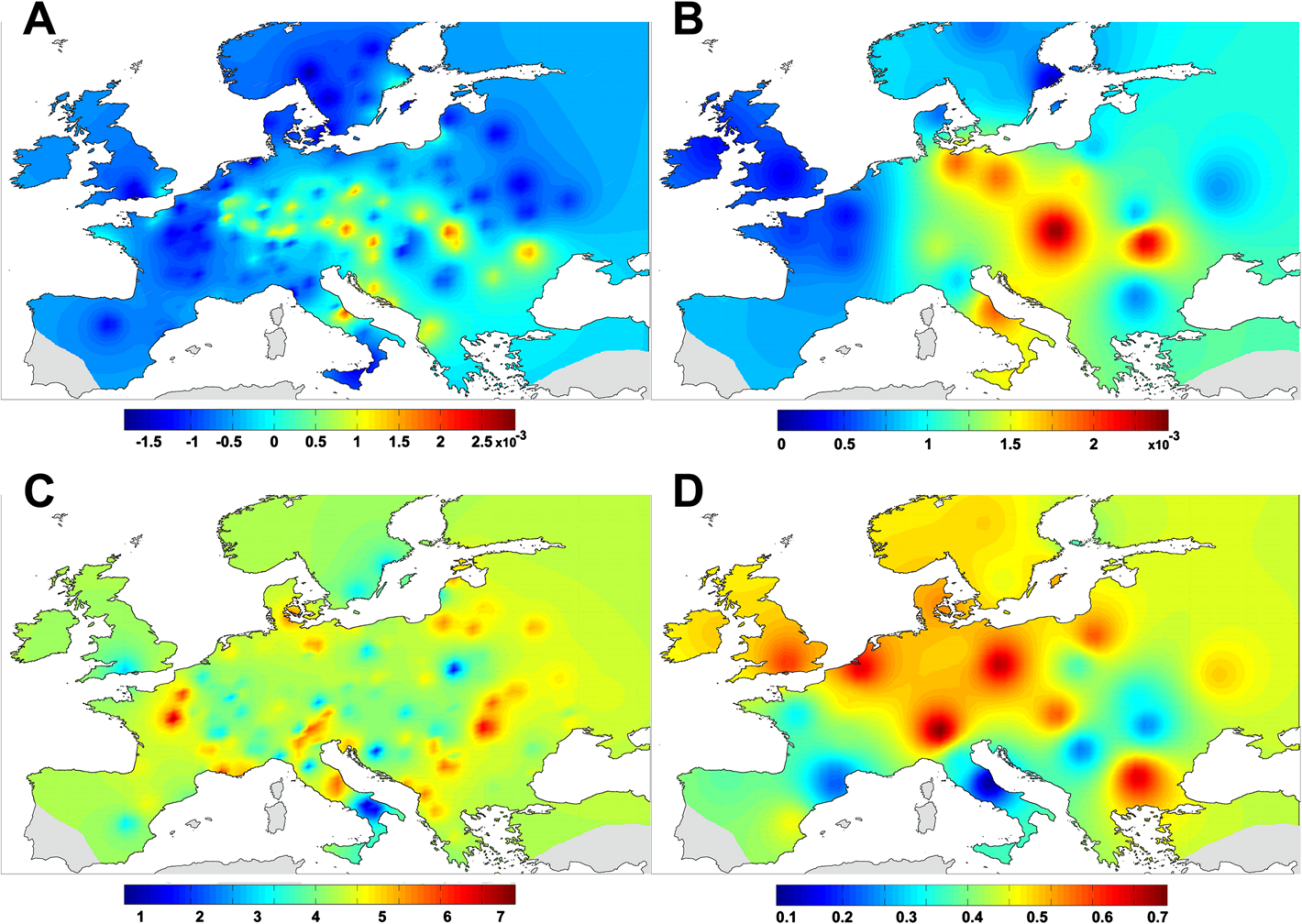
**Divergences, diversity and variability in genetic and MMS in *B. lapidarius*. A**: Interpolation maps of genetic distance. **B**: Interpolation maps of nucleotide diversity. **C**: Interpolation maps of MMS distance. **D**: Interpolation maps of MMS variability.

The interpolation map based on multi-loci genetic distances showed larger local divergence in DNA sequences in sympatric areas between L2b and L3, and L2a and L3 (Central-Eastern Europe and Central Italy, respectively, Figure 3A). Nucleotide and haplotype diversities calculated for each geographic group (Additional file 1, Figure 2) showed: (i) a higher diversity for *COI* in South Italy and Central-Eastern Europe (*h* from 0.521 to 0.8, π from 0.0048 to 0.0063), (ii) higher diversities for *Cytb* in Central-Eastern Europe (*h* from 0.467 to 0.667, π from 0.001 to 0.00171), (iii) a higher diversity for EF1α in Central-Eastern Europe and Italy (*h* from 0.511 to 0.733, π from 0.00161 to 0.00188), (iv) higher diversities for *PEPCK* in Central-Eastern Europe, South Italy and Spain (*h* from 0.456 to 0.8, π from 0.00055 to 0.00132). Interpolation maps based on averaged genetic diversity for all markers confirmed the trend of a larger diversity in South Italy and Central-Eastern Europe (Figure 3B).

### *MMS variation of the red-tailed bumblebee*

#### MMS composition

55 compounds were detected in the MMS of *B. lapidarius* (Additional file 3, Additional file 4). The five main compounds detected were aliphatic compounds: hexadec-9-en-1-ol (C_16_H_32_O), hexadecane-1-ol (C_16_H_34_O), hexadecenoic acid (Δ9 and Δ7, C_16_H_30_O_2_), hexadecenyl hexadecenoate (C_32_H_60_O_2_), and hexadecyl hexadecenoate (C_32_H_62_O_2_). Hexadec-7-enoic acid was only detected in South Italian populations while hexadec-9-enoic acid was detected in other populations. The North Italian populations displayed specific compounds hexadecenal and hexadecanal. The MMS compositions of *B. lapidarius lapidarius* were similar to the previous studies [32,38,39] except for two minor components (tetradecanol and ethyl hexadecenoate).

#### *MMS divergences*

**Figure 4:**
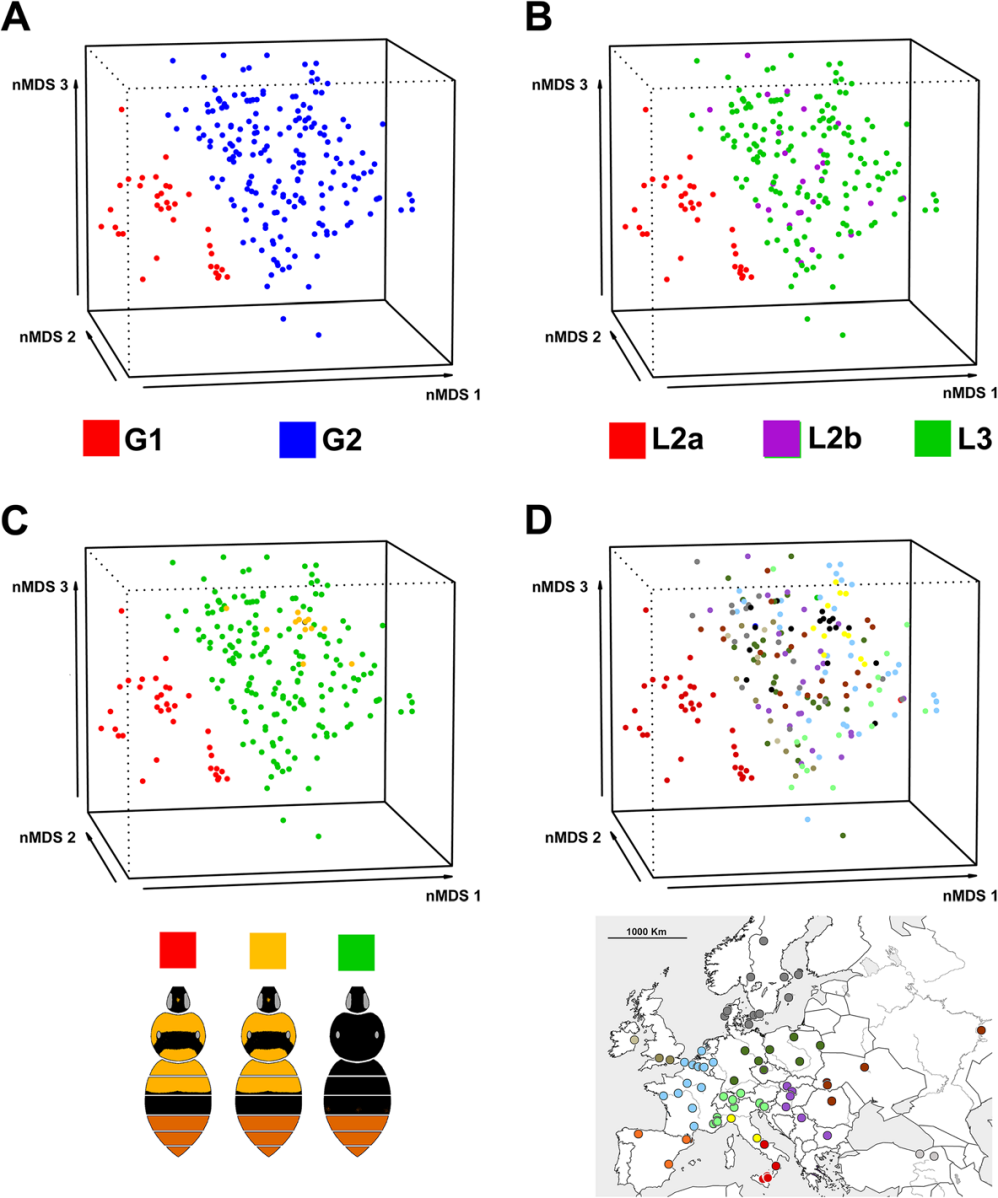
**nMDS analysis of MMS.** Three first axes of the nMDS ordination plot based on Bray-Curtis distances calculated on a male marking secretion matrix of *B. lapidarius* (Stress value = 0.13, R2 = 0.86). **A**: Colour of individuals in nMDS based on groups detected in perMANOVA. **B**: Colour of individuals in nMDS based on genetic lineages (see Figure 1D). **C**: Colour of individuals in nMDS based on colour pattern (in red *B. lapidarius decipiens* South Italy). **D**: Colour of individuals in nMDS based on geographic areas (see Figure 1B).

The nMDS (stress value = 0.13) and perMANOVA analyses separated the European populations into two main groups: Southern Italian populations (group G1) and all other European populations (group G2) (perMANOVA: DF = 1, F = 49.93, p-value < 0.01; Figure 4A). G1 corresponded to the lineage L2a while G2 corresponded to the lineages L2b + L3 (Figure 4B). The IndVal method reveals 18 significant indicator compounds for G1 and seven for G2.

#### *Inter-individual MMS distance and MMS variability*

The interpolation map based on the MMS distance showed larger local divergence in MMS composition in the Alps, Central Italy, Eastern Europe, and Western France (Figure 3C). The MMS variability interpolation map (Figure 3D) revealed (i) the lowest variability in Central Italy, (ii) low variability in West Pyrenees and Carpathian region, and (iii) high variability in NE Europe, the Alps and Central Europe. Comparison of the MMS variability showed significant differences between all geographic groups (AMOVA of multivariate dispersion p-value < 0.01, permutation test of multivariate dispersion p–value < 0.01) and between pairwise geographic groups confirmed these observations (Additional file 5).

### Comparative statistical analyses between geographic origin, colour pattern, genetic and MMS

Mantel tests showed significant positive low correlations between (i) genetic distances *versus* geographic distances (Mantel’s r = 0.18, p-value < 0.01), (ii) MMS distances *versus* geographic distances (Mantel’s r = 0.13, p-value < 0.01), (iii) colour distances *versus* geographic distances (Mantel’s r = 0.32, p-value < 0.01), (iv) genetic distances *versus* MMS distances (Mantel’s r = 0.18, p-value < 0.01), (v) genetic distances *versus* colour distances (Mantel’s r = 0.41, p-value < 0.01), and (vi) MMS distances *versus* colour distances (Mantel’s r = 0.19, p-value < 0.01). Partial Mantel tests showed significant positive low correlations between (i) genetic distance *vs.* MMS distance (Mantel’s r = 0.17, p-value < 0.01), (ii) genetic distances *versus* colour distances (Mantel’s r = 0.38, p-value < 0.01), (iii) MMS distances *versus* colour distances (Mantel’s r = 0.16, p-value < 0.01).

Our correlation analysis showed no linear correlation between genetic diversity and MMS variability (p-value = 0.25).

## Discussion

### Phylogeography of the red-tailed bumblebee

Phylogeographic structure is usually consistent with long-term isolation in multiple refugia during climatic oscillations (e.g. [5]). In this way, the Quaternary glacial events have been shown to explain the intraspecific genetic pattern of many European species [4,15,16]. During Ice Ages, the ranges of most European temperate species contracted to the southern peninsulas of Iberia, Italy and Balkans (classic theory: e.g. [5]). Glaciers, European mountains or repeated marine-flooding during the Pleistocene have constituted major vicariant barriers between these southern refugia leading to allopatric genetic divergence in many species (e.g. [16,40]). Interglacial and postglacial recolonizations of Europe have arisen from these Mediterranean glacial refugia and principally from the Balkans [16,41]. Therefore, it is to be expected that populations of temperate species surviving in Mediterranean refugia display higher genetic variability in the southern peninsulas [41].

Here, our results (trees, networks, Mantel tests, and SAMOVA) detect an intraspecific phylogeographic structure within *B. lapidarius* with three major lineages (L1, L2, and L3; Figure 1; Additional file 2). The roughly estimated molecular clock analyses date divergences between European lineages (L2 and L3) to the Quaternary while the divergence of East Turkish populations takes place earlier (Pliocene). Therefore, the divergence of European lineages can be considered as a consequence of allopatric differentiation during Ice Ages.

**Figure 5:**
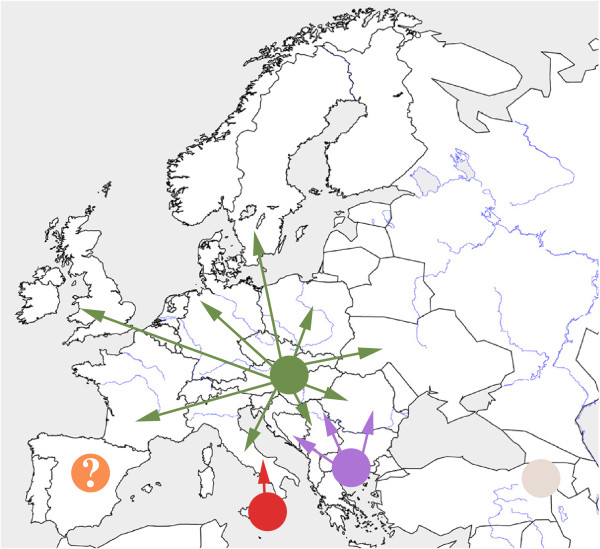
**Scenario of the recent history of *B. lapidarius*.** Approximate location of European refugia and isolated East Turkish populations. The light grey area: range of East Turkish populations. The red area: approximate location of the South Italian refuge. The purple area: approximate location of the refuge of the Balkan region. The green area: approximate location of the refuge of Central-Eastern Europe. The orange area with question mark: the putative Iberian refuge. Arrows are postglacial movements after the Last Ice Age.

A previous study based on morphology has hypothesized that *B. lapidarius* has survived up to the last Ice Age in Iberia, Italy, Balkans, and Turkey prior to a post-glacial recolonization of Europe from the Balkans [42]. Our results suggest an alternative scenario of the recent history of *B. lapidarius* (Figure 5).

The four lineages seem to arise from four regions: East Turkey, South Italy, the Balkan region, and Central-Eastern Europe (C.E. Europe). (i) East Turkey hosts largely differentiated and long-term divergent populations (Figure 1; see molecular clock results). These populations do not contribute to the European genetic pool. (ii) South Italy has acted as a refugium as shown by its large genetic diversity (Figure 3B) and its endemic haplotypes gathered in one distinct lineage L2a (Figures 1, 2 and 3B, Additional file 1). (iii) The Balkan region has acted as a refugium according to its specific haplotypes gathered in one distinct lineage L2b (Figure 1, Additional file 1). The low genetic diversity of the Balkan region could be due to repeated bottlenecking of populations in this region and/or a prolonged bottleneck during past glacial oscillations (e.g. [4,43]). (iv) C.E. Europe has a large genetic diversity (Figure 3B) and many distinct haplotypes gathered in one specific lineage L3 (Figures 1, 2 and 3B, Additional file 1). Even if the genetic diversity is increased by admixture with an allopatric lineage L2b (Figures 1, 3A; see in other species [43]), C.E. Europe hosts the largest diversity of specific haplotypes (Figures 1, 2, 3B). Therefore, this region seems to have been a major glacial refuge for *B. lapidarius*. This evidence fits well with the theory of cryptic refugia in Central, Northern or Eastern Europe, an alternative to the classic theory, observed in other temperate species (review in [44]).

The status of Iberia remains confused according to the slight differentiation/low diversity in mitochondrial markers and the higher differentiation/high diversity in nuclear markers of Iberian populations (similar to South Italian, East Turkish or South East European populations; see Figure 2). The poorly resolved relationship with the lineage L3 (Figure 1) makes it impossible to know if Iberia acted as a refugium (with a strong bottleneck) or has been secondarily colonized by populations from C.E. Europe.

European refugia have probably been isolated by Alpine glaciers, the Alps, Carpathians, Dinaric Alps or repeated marine-flooding of the Crati-Sibari plain (North Calabria, [45]) during the Pleistocene period leading to allopatric divergences of European lineages. In contrast, the frequent drops in the level of the Adriatic Sea during the Quaternary Ice Ages [46] probably allowed gene flow between the Balkans and Italian Peninsula leading to a close relationships between these lineages as observed in rodents (e.g. [47]) and in turtles (e.g. [48]). According to our results, most of Europe has been recolonized from the C.E. Europe at the end of the last Ice Age (Figure 1). The C.E. European lineage has experienced allopatric differentiation (Figure 1C-D; SAMOVA results) probably by reduction of the gene flow [49] resulting from natural sea barriers separating the British Isles and Gotland. The Balkan lineage also dispersed from its refugium and largely mixed with the C.E. European lineage in Central Europe (Figures 1, 3A), a common suture zone in many species (review in [50]). In contrast the South Italian lineage did not contribute to the postglacial colonization, and geographically slightly overlapped the C.E. European lineage in a narrow area in Central Italy (a similar contact zone exists for amphibians and reptiles [51,52]). This was most likely caused by the invasion of North Italy by C.E. European lineage and a competitive exclusion process [53,54] between lineages in this region.

### MMS differentiation

Reproductive traits are generally assumed to be mainly shaped by (i) intraspecific interactions to maximize encounter rates among conspecific mates (sexual selection; [55,56]) and (ii) interspecific interactions to maintain isolation barriers and decrease the likelihood of hybridization events among syntopic sister species [21,57,58]. This results in a stabilizing selection on reproductive traits to promote a species-specific signal (e.g. [21,58]). Across species distribution, local reproductive trait variations (e.g. in moths: [59], in flies: [60], in solitary bees: [28], in birds: [61]), promoted by selection for specific optimized reproductive traits [58,62], can appear due to changes in factors that affect communication systems: (i) mutation of genes involved in reproductive traits (e.g. [62,63]), (ii) intraspecific interactions like local preferences of the receivers [23,64], (iii) the presence/abundance of species with a similar courtship signal which would result in selection for releasers with the most distinct, optimized reproductive traits (e.g. [58]). These changes in factors that affect communication can happen through allopatric differentiation as observed in insular bumblebees where genetic divergence (fostered by geographic isolation) and local specific sexual selection (female preferences, e.g. [34]) lead to MMS divergence from other populations [23]. This reproductive trait differentiation can persist in secondary sympatry if the differentiation has led to the establishment of a reproductive (pre-zygotic) isolation barrier (allopatric hypothesis, see [65]). Alternatively, reproductive trait divergence can happen when formerly isolated lineages meet as a result of range expansion at a secondary contact zone (reinforcement hypothesis; see [25,66,67]). Reinforcement leads to local adaptations in secondary contact zones (e.g. [68,69]).

Our statistical analyses (Figure 4, perMANOVA results) support MMS quantitative as well as qualitative differentiations of Southern Italian populations from other European populations of *B. lapidarius*. These qualitative changes (e.g. from hexadec-9-enoic acid in Southern Italian individuals to hexadec-7-enoic acid in other individuals) have probably arisen from enzyme change (e.g. from Δ9-desaturase to Δ7-desaturase acting on palmitic acid) by mutations of enzyme genes (e.g. [62]) or by activation of a non-functional enzyme gene transcript present in a common ancestor, as observed in moths [64].

The MMS differentiation is poorly explained by geographic distance but displays an obvious geographic pattern (Figure 4, Mantel tests results). The MMS divergence is also weakly correlated with the genetic distance but strongly matches the divergence of the lineage L2a (Figure 4B; Mantel tests results). This suggests that the South Italian MMS differentiation is related to the divergence of the South Italian lineage. Moreover, the lack of gene flow without an obvious geographic barrier between Northern and Southern Italy (Figures 1, 2, 3A, but see [30,42] for putative hybrid records) suggests a reproductive isolation fostered by species intrinsic isolation mechanisms, probably by MMS differentiation leading to pre-zygotic isolation barriers between populations. Therefore we expect that the MMS divergence has appeared subsequent to (i) allopatric divergence during Ice Ages and persistence during current interglacial period (allopatric hypothesis) or (ii) a reinforcement process during the range expansion and the admixture with the C.E. lineage (reinforcement hypothesis). The Southern Italian MMS differentiation seems due to allopatric divergence during glacial isolation rather than reinforcement in secondary contact zone. This is because the Southern Italian MMS pattern is spread over the whole area of the Southern Italian lineage while reinforcement should mainly lead to local adaptations in secondary contact zones as observed in flies [68].

Our statistical analyses demonstrate that the intrapopulational MMS variability is not correlated to the intrapopulational genetic diversity. The geographic areas of high/low intrapopulational MMS variability do not match with areas of high/low intrapopulational genetic diversity (Figure 3B-D). The MMS variability map shows the lowest intrapopulational MMS variability in the contact zone between C.E. European and Southern Italian lineages. We hypothesise that this local decrease of intrapopulational MMS variability could be due to the intensification of the stabilizing selection on MMS for local specific optimized reproductive traits further to new interaction between closely related taxa (lineage L2a and L3) during the postglacial range expansion. This local reinforcement process is also supported by the divergence of Northern Italian populations of C.E. European lineage from other C.E. European lineage populations in MMS composition which increases the MMS divergence with Southern Italian populations (Additional file 3).

The comparisons of the phylogeographic patterns, MMS pattern distribution and MMS variability distribution suggest that the MMS divergence leading to pre-zygotic isolation between Southern Italian and C.E. European lineages has been fostered by past allopatry between refugia and persists in the current interglacial period (allopatric hypothesis). The post-glacial range expansion of these lineages differentiated in MMS seems to have led to a reinforcement process in the secondary contact zone. Further studies in contact zones (e.g. testing the fitness of hybrids, bioassays) are needed to check this hypothesis.

### Colour pattern variation

The genetic distances and lineages only partially reflect the colour patterns [30] (Figure 1, see also Mantel test results). The colour pattern *B. lapidarius caucasicus* fits with L1 while other colour forms (*B. lapidarius decipiens* and *B. lapidarius lapidarius*) spread in L2 and L3 (Figure 1). The MMS distance is also poorly correlated to the colour pattern distance. The MMS and colour patterns are only congruent for the South Italy individuals (Figure 4C). This incongruence between colour patterns, MMS and phylogeny should be a consequence of local Müllerian mimicry processes (e.g. convergence of Spanish *B. lapidarius* to the Pyrenean colour pattern [70]) as observed in other bumblebees [71,72].

### Speciation

The consequence of genetic and MMS divergences between populations fostered by species range contraction during climatic oscillations can range from simple regional variations (e.g. [73]), to a speciation processes between allopatric refugia (e.g. [16,74]). Several pieces of evidence strongly suggest an incipient speciation between Southern Italian and other European lineages of *B. lapidarius* contrary to the current literature (e.g. [30]): (i) the concordance of genetic divergence in tree topologies derived from mitochondrial and nuclear genes [4,75], (ii) the persistence of the genetic divergence through time despite the current secondary contact zone [75], (iii) the divergences in chemical reproductive traits (e.g. qualitative changes or double-bond position shifts) which can lead to discrimination by receivers and to pre-mating isolation [21] as observed in moths (e.g. [64]) and in bumblebees [76]. Therefore, Southern Italy could be a speciation centre for *B. lapidarius* as observed in amphibians [52,77,78] or in reptiles (e.g. [48,51,79]). In the same way, the large mitochondrial and nuclear divergences observed in East Turkish *B. lapidarius* strongly suggest that they are a different species (MMS data not available). In contrast, the speciation process has not happened in other lineages according to current gene flow and the lack of MMS and morphological differentiations. However, these speciation hypotheses need to be checked with further integrative taxonomy, including morphologic, chemical, ethologic and genetic criteria (e.g. [80]) based on an adapted species concept [81] in order to assess the taxonomic status within the *B. lapidarius* complex.

## Conclusions

Quaternary climatic oscillations seem to have led to the European *B. lapidarius* population contraction in three main refugia. These refugia were localized inside Mediterranean peninsulas (Southern Italy and the Balkan) and in Central-Eastern Europe. Inside the Southern Italian refugium, populations have experienced allopatric MMS differentiation and an incipient speciation process from other lineages. At the end of Ice Age, the range expansion of lineages have led to secondary contact zone between the C.E. Europe lineage and Southern Italy lineage where a reinforcement process on local MMS patterns seems to take place.

This study suggests that the population movement during Quaternary climatic oscillations can lead to divergence in reproductive traits by allopatric differentiation during Ice Age and by reinforcement during post-glacial recolonization.

## Methods

### Sampling

259 males of *B. lapidarius* covering its whole European distribution were collected (Figure 1A, Additional file 6). Males were killed by freezing at -20°C. The MMS were extracted in 400 μl of hexane from dissected cephalic labial glands or entire cut heads [82]. Bodies without heads were preserved in 99% ethanol for DNA extraction. All samples were stored at -40°C until analyses. Populations from the Caucasus (*B. lapidarius eriophorus*) and Morocco (*B. lapidarius atlanticus*) were not collected. Closely related species were also collected as outgroup [83] (see Additional file 6).

### Genetic analyses

#### *Gene selection*

We sequenced five genes commonly used to study interspecific and intraspecific relationships (e.g. [80,83,84]: mitochondrial cytochrome oxidase 1 (*COI*), cytochrome b (*Cytb*), nuclear protein-coding genes long-wavelength rhodoposin copy 1 (*LWhH*), elongation factor-1 alpha F2 copy (*EF-1α*), and phosphoenolpyruvate carboxykinase (*PEPCK*). We used a multi-gene approach to check congruence of phylogenetic and phylogeographic patterns inferred from independent genes.

#### *DNA preparation, amplification and sequencing*

Total DNA was extracted using a QIAGEN DNeasy® Tissue Kit (Quiagen Inc., Valencia, CA). Legs were removed from the specimen, crushed using liquid nitrogen, and digested (four hours in proteinase K at 56°C). Polymerase chain reaction (PCR) amplifications were carried out for all genes and all samples using primer pair Apl2013/ApH2931 [84] for *COI*, CB1/CB2 [85] for *Cytb*, LWRhF/LWRhR [86] for *LWRh*, F2-ForH/F2-RevH2 [87] for *EF-1α*, and FHv4/RHv4 [83] for *PEPCK*. PCR amplifications were carried out by initial denaturing for three minutes at 94°C, 35 (*COI*, *LWRh*, *EF-1α*) or 40 (*Cytb* and *PEPCK*) cycles of one minute denaturing at 94°C, one minute annealing at 51°C (*COI*), 50°C (*Cytb*), 60°C (*LWRh*), 54°C (*EF-1α*) or 48.5°C (*PEPCK*), two minutes elongation at 72°C and a final extension for ten minutes at 72°C. Voucher specimens and PCR products used in molecular investigation were deposited at the University of Mons (Belgium). Genes were sequenced with an ABI 3730XL sequencer (Applied Biosystems, Foster City, CA, USA), with ABI 3730 DNA analyzer or by GENOSCOPE (*Centre National de Séquençage*; Evry, France). Both strands of each PCR product were sequenced. Consensus sequences were computed with CodonCode Aligner 3.0.1. There is no uncertainty in the consensus sequences. The bumblebee origin of each sequence was checked with BLAST 2.2.20 [88]. The alignment was performed by MAFFT ver.6. (using FFT-NS-2 algorithms, default parameters) [89]. The data matrix was computed on Mesquite 2.74 (build 486) [90]. Translation to proteins (using the *Drosophila* mitochondrial DNA genetic code or Universal genetic code) was performed on Mesquite. Sequences were deposited in GenBank [GenBank: KC915396 to KC916646] (Additional file 6).

#### *Haplotype relationships*

We performed phylogeographic and phylogenetic analyses to investigate the relationships between haplotypes and to infer the phylogenetic lineages. A test of saturation was applied to each gene fragment in PAUP* 4.0b 10 [91].

Haplotype networks were computed using the me-dian-joining method (MJM, [92]) in Network 4.6.1.0 (http://www.fluxus-engineering.com) for each gene. The median-joining method uses a maximum parsimony approach to search for all the shortest phylogenetic trees for a given data set [92]. To reconstruct the network, we weighted transversions twice as high as transitions.

Phylogenetic analyses were carried out using distance (neighbour joining [NJ]), maximum likelihood (ML) and Bayesian (MB) methods. Trees were rooted with species from outgroup. For each approach, we analysed (i) each genes individually, (ii) all mitochondrial genes combined (*COI* + *Cytb*), (iii) all nuclear genes combined (*EF-1α* + *LWRh* + *PEPCK*), and (iv) all genes combined. We assessed the level of incongruence in phylogenetic reconstructions among genes by comparing congruence and incongruence of well-supported clades across NJ, ML and MB trees of each gene. Overall, the gene trees were not strongly conflicting with each other and well-supported clades were similar across all trees (see supplementary trees at [TreeBASE ID: S14299]). We did not perform the incongruence length difference test [93] because its utility in evaluating homogeneity test has been questioned (e.g. [94]).

NJ analyses were performed using PAUP*. We used jModeltest [95] using the Akaike information criteria corrected for small sample sizes (AICc, [96]) to determine the best fitting substitution models (GTR + G). The robustness of the NJ trees was assessed by bootstrap resampling (10,000 random replications, [97]).

ML analyses were conducted in GARLI 2.0 [98]. Each gene was partitioned to explore the best substitution model: (i) *EF-1α* into two exons and one intron, (ii) *LWRh* into three exons and two introns, (iii) *PEPCK* into two exons and two introns, and (iv) *COI*, *Cytb* and each nuclear exon by base positions (1^st^, 2^nd^ and 3^rd^). The best fitting substitution models were chosen with jModeltest using the AICc for each dataset. The chosen models were: (i) for *COI*: TIM1 + G (1^st^), F81 (2^nd^) and TPM1uf + G (3^rd^); (ii) for *Cytb*: TrN + I + G (1^st^), F81 (2^nd^) and HKY + G (3^rd^); (iii) for *EF-1α* exon 1: F81 (1^st^ and 2^nd^) and HKY (3^rd^); (iv) for *EF-1α* intron: HKY; (v) for *EF-1α* exon 2: F81 (1^st^), K80 (2^nd^) and TPM3 (3^rd^); (vi) for *LWRh* exon 1: F81 (1^st^ and 2^nd^) and K80 (3^rd^); (vii) for *LWRh* introns 1 and 2: F81; (viii) for *LWRh* exon 2: F81 (1^st^), JC (2^nd^) and K80 (3^rd^): (ix) for *LWRh* exon 3: JC (1^st^, 2^nd^ and 3^rd^); (x) for *PEPCK* introns 1 and 2: HKY; (xi) for *PEPCK* exon 1: TrN (1^st^), JC (2^nd^) and TrNef (3^rd^); (xii) for *PEPCK* exon 2: F81 + I (1^st^ ), K80 (2^nd^) and JC (3^rd^). A random starting tree and the automated stopping criterion (stop when the ln score remained constant for 20,000 consecutive generations) were used. Ten independent runs in GARLI were carried out; the topology and –ln(L) were identical among replicates. The highest likelihood of one of those runs was retained. Statistical confidence in nodes was evaluated using 10,000 non-parametric bootstrap replicates [97] using the automated stopping criteria set at 10,000 generations. Topologies with bootstrap values ≥70% were considered well supported [99].

MB analyses were carried out using MrBayes 3.1.2 [100]. The model selection process was the same as that for ML analyses. Selected models which are not implemented in MrBayes were substituted by the closest overparameterized model [101]. The TIM1, TIM1uf, and TrN were replaced by the GTR model whereas the TPM3 and TrNef were replaced by the SYM model. The proportion of invariant sites (I) and gamma distributed rates (G) defined in jModeltest were conserved in all models. Five independent analyses were carried out (30 million generations, four chains with mixed-models, default priors, saving trees every 100 generations). The analyses were stopped after checking convergence between runs using the average standard deviation of split frequencies and by plotting likelihood values across generations using Tracer 1.4 [102]. The first three million generations were discarded as burn-in. The phylogeny and posterior probabilities were then estimated from the remaining trees and a majority-rule 50% consensus tree was constructed. Topologies with posterior probabilities ≥ 0.95 were considered as well supported [103].

#### *Estimating divergence time*

Following the approach of Duennes *et al. *[104], we analyzed the *COI* dataset in BEAST v1.7.2 [105] to roughly estimate the time to most recent common ancestor of *B. lapidarius* haplogroups. Using the GTR + G model selected by jModeltest, we ran Markov chain Monte Carlo simulations with the coalescent constant population size tree model and the strict clock model. Prior, the relaxed uncorrelated lognormal molecular clock model [106] was implemented to assess the clock-like nature of the data. The sampled marginal posterior probability (PP) distribution of both standard deviation and coefficient of variation of substitution rates among tree branches included 0 in the 95% HPD, indicating that there was no strong evidence for substantial rate heterogeneity among lineages [105]. We specified a range of possible substitution rates which include extreme rate of insect mitochondrial genes recorded in the literature (see [104]) using a flat prior ranging from 1×10^-9^ to 1×10^-7^ substitutions site^-1^ and year^-1^. Simulations were run for 30 million generations, sampling every 1000 generations. Four independent runs were assessed in Tracer v1.4.1 [102] to confirm convergence, determine burn-in, and examine the effective sample size of all posterior parameters. Log files from each run were combined in LogCombiner v1.6.1 [102] for final parameter estimates.

#### *Identifying population structure*

Population structure was assessed for each gene by estimating “global” Φ_ST_ statistic on populations (i.e. only AMOVA Φ_ST_ estimator when considering only one group of populations [107]) using ARLEQUIN 3.5 [108] with 100,000 permutations. When global Φ_ST_ statistic was significant for one gene, we analyzed the population structure using the spatial AMOVA procedure (SAMOVA) [109] implemented in SPADS 1.0 [110]. This procedure assigns populations to groups based on geographical vicinity and sequence similarity. The most likely structure corresponds to the partition of populations that maximized among-group variation measured by the AMOVA Φ_ST_ statistic [107]. We performed ten independent repetitions of 10,000 simulated annealing steps for *K* values varying from two to 45.

#### *Estimating genetic diversity*

Genetic diversity was estimated with the number of different haplotypes, the haplotype diversity (h, [111]), and the nucleotide diversity (π, [112]) using DnaSP [113] within defined geographic groups and for each gene separately. The defined geographic groups gathered samples from the same geographic regions (see Additional file 6).

#### *Cartography of inter-individual genetic distance and intra-population genetic diversity*

We used an extension of the method developed by Miller [114] to represent spatial variations of genetic patterns (distance and diversity). The method of Miller [114] is based on a connectivity network (e.g. a Delaunay triangulation) built from the sampling localities. Inter-individual genetic distances are first estimated and assigned to landscape coordinates at midpoints of each Delaunay triangulation edge. An interpolation procedure (inverse distance-weighted interpolation, [115,116]) is then used to infer genetic distances at locations on a uniformly spaced grid. Here, we also used this method to represent the spatial variation of any kind of distance and measures of diversity. The interpolation procedure of measures of diversity is based on (diversity) values directly estimated at each sampling point rather than on (distance) values assigned at midpoints of each edge of a connectivity network. Interpolation surfaces were generated with the MATLAB (The MathWorks, Inc) functions GDisPAL for “genetic distance patterns across landscapes” and GDivPAL for “genetic diversity patterns across landscapes” [110]. All generated surfaces were finally superimposed on a map of the distribution area of the geographic region studied. We generated two surfaces based (i) inter-individual genetic distance measured as DNA sequence mismatches averaged over the four polymorphic loci (i.e. averaged p-distances over loci), and (ii) on genetic diversity measured with the nucleotide diversity estimated within each geographic group (see Additional file 1) and averaged over the four polymorphic loci Both the inter-individual genetic distance and nucleotide diversity were computed with SPADS [110]. The geographic coordinates of geographic groups were the barycenter between all sampling places included in geographic groups. All surfaces were generated using a distance weighting parameter *a* = 2 and distance surfaces were both based on a Delaunay triangulation connectivity network. For the interpolation parameter *a*, we selected a value that allowed to generate interpolation surfaces displaying local details. As advised by Miller et al [114], we performed the distance interpolations using (i) residual distances derived from the linear regression of genetic *vs.* geographical distances [117] and (ii) MMS *vs.* geographic distances in order to account for potential correlation between genetic and geographic distances.

### MMS analyses

#### *Chemical analyses of MMS*

We analyzed the MMS of 226 individuals (Additional file 6). The composition of MMS was determined by gas chromatography-mass spectrometry (GC/MS) on a Finigan GCQ with a DB-5 ms non-polar capillary column (5% phenyl (methyl) polysiloxane stationary phase; 30 m × 0.25 mm × 0.25 μm) and an ion trap in electron impact mode “full scan (300-600)”. We used a splitless injection mode (220°C) and helium as carrier gas (50 cm/s). The temperature programme of the column was set to 70°C for two minutes and then increased at a rate of 10°C/min to 320°C. The temperature was then held at 320°C for five minutes. Compounds were identified in Xcalibur™ using their mass spectra compared to those at National Institute of Standards and Technology library (NIST, U.S.A) using NIST MS Search 2.0. The double bond positions were determined i) from mass spectra of dimethyl disulphide adducts of unsaturated components (reaction time: four hours) and ii) by chemical ionization with acetonitrile as a reaction gas. The products were analyzed by GC/MS using the same temperature programme as for the original extracts. An ion trap GC/MS instrument (Varian Saturn, 2000) was used for chemical ionization. The compound relative quantification of MMS was estimated using a gas chromatograph Shimadzu GC-2010 with a SLB-5 ms non-polar capillary column 5% diphenyl/95% dimethyl siloxane; 30 m × 0.25 mm × 0.25 μm) and a flame ionization detector. The chromatographic conditions were the same as above. The absolute amounts of compounds were quantified using GCsolution Postrun (Shimadzu Corporation) with automatic peak detection and noise measurement. Relative amounts (RA in%) of compounds in each sample were calculated by dividing the absolute amounts of compounds by the total absolute amount of compounds in each sample. We did not use any correction factor to calculate the RA of individual compounds. All compounds for which RA were recorded as less than 0.1% for all specimens were discarded [118]. The data matrix for each species was elaborated with the relative proportion of each compound for each individual [82] using GCAligner 1.0 [119] before final check.

#### *Comparative statistical analyses based on MMS*

All statistical analyses were performed using R [120] to detected MMS differentiations between populations. Data consisting of the relative proportion of all compounds were transformed (log (x-1)) to reduce the large difference of abundance between highly and slightly concentrated compounds and standardized (mean = 0, standard deviation = 1) to reduce the sample concentration effect [82]. We compared the profile for each sample (ungrouped) with non-metric multidimensional scaling (nMDS) ordination using a Bray-Curtis similarity matrix, three dimensions and 50 runs (R-package ecodist, [121]). When we detected MMS differentiations between population groups in nMDS, we assessed results by performing permutation-based version of the multivariate analysis of variance (perMANOVA) using the Bray-Curtis similarity matrix and 10,000 permutations (R-package vegan, [122]). Like conventional analyses of variances, the perMANOVA calculates an F statistic by taking the ratio of among group sums of squares to within group sums of squares. The perMANOVA is robust to violations of multivariate normality but requires homogeneity of variances. When we tested more than two groups and the returned p-value was significant (p < 0.01), multiple pairwise comparisons were conducted and p-values were adjusted using Bonferroni’s correction to avoid increases of type error I due to multiple testing. Prior to perMANOVA we checked its assumption (similar variance homogeneity between groups) by performing a distance-based test for multivariate homogeneity of group dispersions (MHGH) for a one-way ANOVA design [123]. The procedure first calculates the Euclidean distances between MMS composition and respective geographic group centroids on Pearson r correlation matrices (R- package vegan, [122]). To test if one group was more variable than the other, the magnitudes of these distances were then compared between groups using ordinary ANOVA. Moreover, a permutation test was run to generate a permutation distribution of F under the null hypothesis of no difference in dispersion between groups (R- package vegan, [122]).

#### *Specific MMS compounds*

To determine compounds specific to groups defined by nMDS and confirmed by perMANOVA, we used the indicator value (IndVal) method [124]. A high value is obtained when the compound is specific and regular to a particular group compared to the whole set of observations. The statistical significance of a compound as an indicator at the 0.05 level was evaluated using a randomization procedure.

#### *Chemical variability within populations*

We compared variability of MMS between each geographic group (pairwise analyses) using a distance-based test for MHGH for a one-way ANOVA design [123].

#### *Cartography of inter-individual MMS distance and intra-population MMS variability*

We used the extended Miller’s [114] method (i.e. GDisPAL and GDivPAL functions, see above) to represent spatial variations of MMS patterns (distance and variability). We generated two different surfaces: (i) on MMS distance (Bray-Curtis similarity matrix with dataset transformed and standardized), (ii) on MMS variability (the mean Euclidean distance between MMS composition of each sample and its respective geographic group centroids (computed on Bray-Curtis similarity matrix with dataset transformed and standardized)). MMS variability was estimated within each geographic group (see Additional file 1). The geographic coordinates of geographic groups were the same as genetic analyses. All surfaces were generated using a distance weighting parameter *a* = 2 and distance surfaces were both based on a Delaunay triangulation connectivity network. Furthermore, we performed the distance interpolations using residual distances derived from the linear regression of MMS *versus.* geographical distances in order to account for potential correlation between MMS and geographical distances.

### Comparative statistical analyses between geographic origin, colour patterns, genetic and MMS

We investigated correlations between genetic divergence, colour patterns, MMS differentiation, and geographic distance by performing six Mantel tests [125] with 10,000 random permutations in R (R-package vegan): (i) genetic distances *versus* geographic distances, (ii) MMS distances *versus* geographic distances, (iii) colour distances *versus* geographic distances, (iv) genetic distances *versus* MMS distances, (v) genetic distances *versus* colour distances, and (vi) MMS distances *versus* colour distances. Geographic distances between all samples were computed with Geographic Distance Matrix Generator version 1.2.3 [126]. Genetic distances were calculated in the same way as for the cartography of inter-individual genetic distance (see above) and were thus based on DNA sequence mismatches averaged over the four polymorphic loci (i.e. averaged p-distances over loci) computed with SPADS [113]. The MMS distance matrix was the individual-by-individual Bray-Curtis distance matrix based on MMS compounds. The colour distance matrix compared the colour pattern between all individuals and was generated as followed: when two individuals shared the same colour pattern, their colour distance was set to “0” and otherwise it was set to “1”.

We also performed three partial Mantel tests based on 10,000 random permutations controlling for spatial autocorrelation between (i) genetic distances *versus* MMS distances, (ii) genetic distances *versus* colour distances, and (iii) MMS distances *versus* colour distances.

We used a generalized linear model [127] to estimate the potential linear correlation between genetic diversity and MMS variability. To estimate diversities we gathered samples from the same geographic groups (Additional file 6) and excluded geographic groups with only one sample. Genetic diversity used was the nucleotide diversity estimated within each geographic group and averaged across loci. MMS variability was the mean Euclidean distance between MMS composition of each sample and its respective geographic group centroids (computed on Bray-Curtis similarity matrix with dataset transformed and standardized).

### Data availability

The genetic datasets supporting the results of this article are available at TreeBase http://purl.org/phylo/treebase/phylows/study/TB2:S14299. Genbank accession numbers are listed in the Additional file 6 - Table of sampling. The MMS dataset supporting the results of this article is included within the article and its Additional file 3.

## Abbreviations

AMOVA: Analysis of molecular variance; C.E. Europe: Central and Eastern Europe; h: Haplotype diversity; L1: East Turkey lineage; L2: Southern Italy (L2a) and South Eastern European (L2b) lineages; L3: C.E. European lineage; LWRh: Nuclear protein-coding genes long-wavelength rhodoposin copy 1; MB: Bayesian; MHGH: Multivariate homogeneity of group dispersions; ML: Maximum likelihood; MMS: Male marking secretion; NJ: Neighbour joining; perMANOVA: Permutation-based version of the multivariate analysis of variance; SAMOVA: Spatial AMOVA; π: Nucleotide diversity.

## Competing interests

The authors declare that they have no competing interests.

## Authors’ contributions

TL and SD conceived the study and interpreted the results. The experiments were designed by TL. TL, SD, MV, and IV analysed the data. TL, SD, DM, PL, IV, J-YR, and PR contributed to materials/analysis tools. TL wrote the manuscript. All authors finalised the manuscript. All authors read and approved the final manuscript.

## Supplementary Material

Additional file 1 **MMS variability, nucleotide diversity and haplotype diversity observed among geographic groups.** Geographic group: see Additional file 1, MMS variability: Euclidean distance between MMS composition of each sample and its respective geographic group centroids. Click here for file

Additional file 2 **The results from the SAMOVA analyses. K: number of genetic clusters.** Number in gene column is the group of each geographic group. Click here for file

Additional file 3 **MMS data matrix (relative amounts of each compound) of *B. lapidarius.***
Click here for file

Additional file 4 **List of the identified compounds in European *B. lapidarius.*** Molecular weight [MW (m/z)], median [Med (%)], first and fourth quartiles [Q1 (%) and Q2 (%)], minimum and maximum [Min (%) and Max (%)] of the 55 identified compounds. Unknown x are undetermined compounds. Click here for file

Additional file 5 **The results of AMOVA of multivariate dispersion test between each geographic group.** Values are the p-values. Click here for file

Additional file 6 **Table of sampling.** Sample Code: sample labels used in analyses and supplementary tree, Taxa: Name of taxa, Geographic groups: Groups of sampling place, *COI*, *Cytb*, *EF-1α*, *LWRh*, and *PEPCK* are the Genbank accession numbers for each sample. Click here for file
